# Thymidine Phosphorylase Inhibitory Potential and Molecular Docking Studies of Secondary Metabolites Isolated From *Fernandoa adenophylla* (Wall. ex G. Don) Steenis

**DOI:** 10.1002/cbdv.202500449

**Published:** 2025-05-06

**Authors:** Abdur Rauf, Majid Khan, Umer Rashid, Anees Saeed, Zafar Ali Shah, Zubair Ahmad, Abdulhakeem S. Alamri, Walaa F. Alsanie, Imtiaz Khan, Humaira Hussain, Majid Alhomrani, Marcello Iriti

**Affiliations:** ^1^ Department of Chemistry University of Swabi Swabi Khyber Pakhtunkhwa Pakistan; ^2^ College of Chemistry Fuzhou University Fuzhou China; ^3^ Department of Chemistry COMSATS University Islamabad, Abbottabad Campus Abbottabad Pakistan; ^4^ Department of Agricultural Chemistry and Biochemistry The University of Agriculture Peshawar Pakistan; ^5^ Department of Clinical Laboratory Sciences, The Faculty of Applied Medical Sciences Taif University Taif Saudi Arabia; ^6^ Research Center for Health Sciences, Deanship of Graduate Studies and Scientific Research Taif University Taif Saudi Arabia; ^7^ Department of Entomology The University of Agriculture, University of Peshawar Peshawar Pakistan; ^8^ Department of Biochemistry Abbottabad University of Science & Technology Abbottabad Pakistan; ^9^ Department of Biomedical, Surgical and Dental Sciences University of Milan Milan Italy; ^10^ National Interuniversity Consortium of Materials Science and Technology (INSTM) Florence Italy

**Keywords:** *Fernandoa adenophylla*, in silico study, inhibition, thymidine phosphorylase

## Abstract

This study investigates the potential of *Fernandoa adenophylla*, a South American plant, as a reservoir of compounds with thymidine phosphorylase (TP) inhibitory activity. Through a comprehensive approach combining in vitro assays and molecular docking analysis, we isolated and characterized bioactive compounds from *F. adenophylla*, including lapachol, alpha‐lapachone, Peshawaraquinone, dehydro‐α‐lapachone, and indanone derivative (Methyl‐1,2‐dihydroxy‐2‐(3‐methylbut‐2‐en‐1‐yl)‐3‐oxo‐2,3‐dihydro‐1H‐indene‐1carboxylate). Our results reveal substantial TP inhibition by these compounds, with Lapachol (**1**) and Indanone derivative (**5**) demonstrating notable potency, exhibiting IC_50_ values of 2.3 ± 0.1 and 1.8 ± 0.5 µM, respectively. Molecular docking analysis supported experimental in‐vitro results, revealing strong binding affinities of the tested compounds toward both human TP and *Escherichia coli* TP, with the indanone derivatives exhibiting the most favorable binding energies (‐7.50 and ‐7.80 kcal/mol, respectively). Key interactions with important catalytic residues were identified, highlighting these natural products' structural complementarity and binding stability. These docking results correlate well with the observed in vitro inhibitory activities, reinforcing the compounds' therapeutic relevance. This study underscores the therapeutic potential of *F. adenophylla*‐derived compounds as effective TP inhibitors, highlighting the significance of natural products in drug discovery.

## Graphical abstract

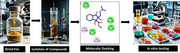

## 1 Introduction

Medicinal plants have been a wellspring of bioactive compounds, contributing significantly to the development of novel therapeutic agents in modern pharmacology [1, 2, 3]. Natural products derived from plants harbor diverse chemical structures and possess a myriad of biological activities, making them invaluable resources for drug discovery and development [4, 5]. The rich biodiversity of plants offers a vast reservoir of phytochemicals, each with its unique pharmacological properties and therapeutic potential [6, 7]. Over the centuries, indigenous cultures worldwide have harnessed the healing properties of medicinal plants, employing traditional remedies to alleviate various ailments and diseases. In recent decades, advancements in phytochemical analysis, bioassay techniques, and molecular pharmacology have facilitated a deeper understanding of the medicinal properties of plants and their bioactive constituents [8, 9]. This has led to an exponential increase in the identification and isolation of pharmacologically active compounds from medicinal plants. These bioactive compounds exhibit a wide range of biological activities, including antioxidant, anti‐inflammatory, antimicrobial, antiviral, anticancer, and immunomodulatory effects [10, 11]. Moreover, the utilization of medicinal plants and their derivatives in drug discovery and development offers several advantages over synthetic compounds. Natural products often possess greater chemical diversity [11, 12]. Among these plants, *Fernandoa adenophylla* stands out as a promising source of bioactive compounds with potential medicinal applications.

The medicinal potential of *F. adenophylla*, a plant indigenous to South America, has long been recognized in traditional folk medicine. Recent scientific research has delved into this traditional knowledge, unveiling the presence of bioactive compounds across different parts of the plant, such as the leaves, stems, and roots [11, 13–15]. Among the numerous bioactivities observed, one of the most promising areas of study involves the inhibition of TP, an enzyme critical in nucleotide metabolism [16].

Despite growing interest in TP inhibitors from synthetic sources, there remains a significant gap in the literature regarding natural inhibitors, particularly those derived from *F. adenophylla*. TP has emerged as a validated therapeutic target in various pathological conditions, including cancer, inflammation, and angiogenesis. However, the exploration of plant‐derived compounds as TP inhibitors is still limited. This highlights the need for systematic studies on phytochemicals with potential TP inhibitory activity, particularly from traditionally used medicinal plants.

Our study addresses this gap by focusing on the isolation and characterization of five specific compounds from *F. adenophylla*, which have not yet been comprehensively studied for their TP inhibitory activity. TP has emerged as a significant target in various pathological conditions, including cancer, inflammation, and angiogenesis. By inhibiting TP activity, researchers hope to develop effective treatments against these diseases [17, 18]. The discovery of TP‐inhibiting properties in *F. adenophylla* suggests its potential as a source of novel anti‐cancer and anti‐inflammatory agents. Further exploration of the mechanisms underlying TP inhibition by compounds found in *F. adenophylla* could pave the way for the development of new therapeutic interventions with potentially significant clinical implications. Additionally, investigating the specific bioactive compounds responsible for TP inhibition in different parts of the plant may lead to the isolation of potent pharmaceutical leads for future drug development efforts [19]. Moreover, in silico studies are increasingly recognized for their crucial role in elucidating the molecular mechanisms underlying the interaction between bioactive compounds and their target enzymes. Computational approaches provide valuable insights into the binding modes, affinity, and structure‐activity relationships of these compounds [12]. By leveraging computational modeling techniques, researchers can better understand how these compounds interact with TP at the molecular level, facilitating the rational design and optimization of TP inhibitors. Integrating in vitro experimental data with computational predictions enhances our understanding of the therapeutic potential of *F. adenophylla*‐derived compounds and aids in the development of more effective TP‐targeted therapeutics.

Therefore, the present study is driven by the following hypothesis: specific phytochemicals isolated from *F. adenophylla* exhibit significant inhibitory activity against TP, thereby offering therapeutic potential against TP‐related diseases. This hypothesis is tested through a combination of experimental and computational approaches. This research paper aims to: (i) isolate and characterize five compounds Lapachol (**1**), Alpha‐lapachone (**2**), Peshawaraquinone (**3**), Dehydro‐α‐lapachone (**4**), and an Indanone derivative (**5**) from *F. adenophylla*, (ii) evaluate their in vitro TP inhibitory activity, and (iii) explore their binding interactions through molecular docking and in silico studies. This integrated approach is expected to yield novel insights into natural TP inhibition and potentially contribute to the development of plant‐based therapeutics targeting TP.

## 2 Results

### 2.1 TP Activity

The in vitro activity of the isolated compounds against TP was evaluated and summarized in Table 1. Among the compounds tested, Lapachol (**1**), Alpha‐lapachone (**2**), Peshawaraquinone (**3**), Dehydro‐α‐lapachone (**4**), Indanone derivative (**5**) exhibited substantial inhibitory activity against TP, with inhibition percentages ranging from 86.2% to 91.3%. These compounds demonstrated promising IC_50_ values, indicating their potency in inhibiting TP activity, with Lapachol (**1**) and Indanone derivative (**5**) showing the lowest IC_50_ values of 2.3 ± 0.1 and 1.8 ± 0.5 µM, respectively. Conversely, dehydro‐α‐lapachone (**4**) exhibited relatively lower inhibitory activity, with an IC_50_ value of 17.2 ± 1.0 µM. Additionally, 7‐deazaxanthine, employed as a standard inhibitor, demonstrated notable inhibition of TP activity, with an IC_50_ value of 15.1 ± 0.1 µM. These findings underscore the potential of the isolated compounds, particularly Lapachol (**1**), alpha‐lapachone (**2**), Peshawaraquinone (**3**), and indanone derivative (**5**), as effective inhibitors of TP enzyme activity.

**TABLE 1:** In vitro activity of the isolated compounds against thymidine phosphorylase.

Compound	% Inhibition	IC_50_ ± SEM (µM)
Lapachol (**1**)	86.2	2.3 ± 0.1
Alpha‐lapachone (**2**)	88.3	3.6 ± 0.9
Peshawaraquinone (**3**)	87.1	4.9 ± 0.8
Dehydro‐α‐lapachone (**4**)	82.6	17.2 ± 1.0
Indanone derivative (**5**)	91.3	1.8 ± 0.5
7‐Deazaxanthine	80.6	15.1 ± 0.1

### 2.2 Mechanism‐based Studies

**FIGURE 2:**
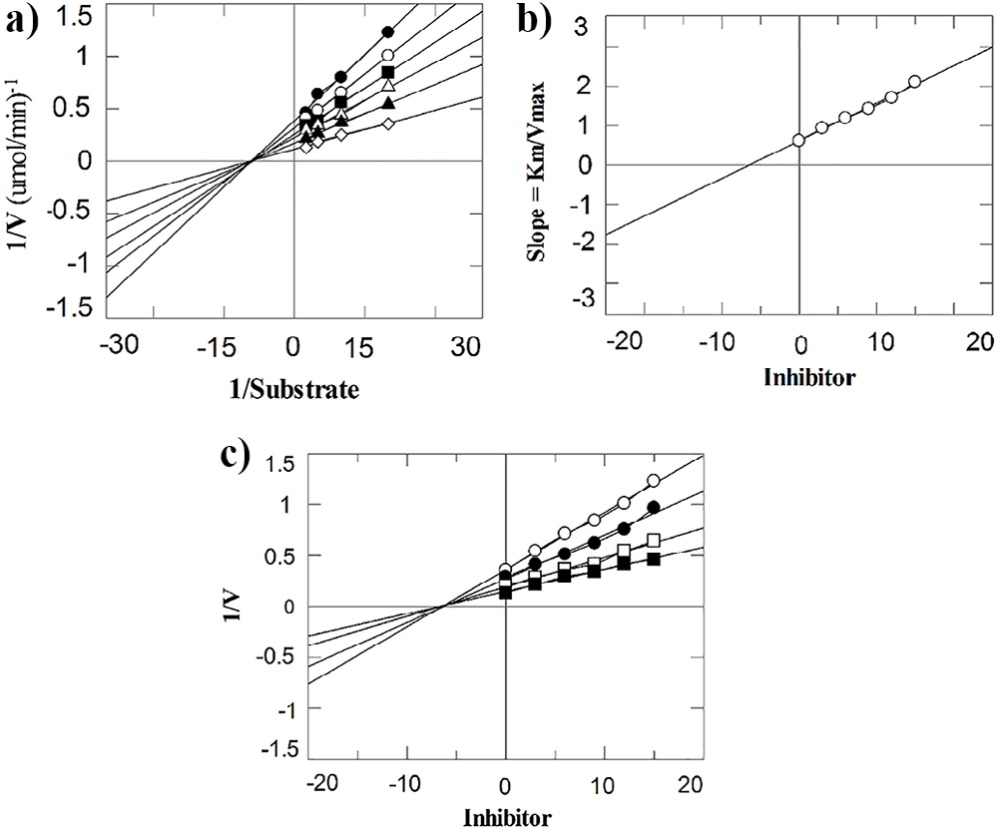
Kinetic mode of compound **2** (**a**) Depicts Lineweaver‐Burk plot of compound **2** in which the reciprocal of substrate concentration (1/S) is plotted on the x‐axis, while the reciprocal of rate of reaction (1/V) is plotted on the y‐axis in the absence and presence of different concentrations of compound **2**. The figure shows that the apparent km of the enzyme remains unaffected while the apparent V_max_ decreases. (**b**) Depicts secondary re‐plot of Lineweaver‐Burk plot between the slopes (km/V_max_) of each line versus different concentrations of compound **2**. (**c**) Depicts Dixon plot of reciprocal of rate of reaction (velocities) versus different concentrations of compound **2**.

Mechanism‐based studies were carried out on the selected compounds, that is, 1, 2, and 5. All the compounds inhibited the enzyme in a non‐competitive manner (Figure 2). These compounds interacted with the allosteric site of the enzyme. As a result of this, the Km value (Michaelis–Menten constant) does not change, however, the Vmax value (maximum velocity) varies from its point concerning compound doses. Therefore, when thymidine is utilized as the variable substrate, these molecules do not interact competitively with the phosphate‐binding sites of TP or thymidine. The dissociation constants (Ki) ranged from 4.6 to 7.1 µM, as determined by secondary replotting the Lineweaver‐Burk and Dixon plots (Table 2).

**TABLE 2:** Mechanism‐based studies of the most active inhibitors of thymidine phosphorylase.

Compound	Ki ± SEM	Type of Inhibitor
1	4.6 ± 0.006	Non‐competitive
2	4.9 ± 0.008	Non‐competitive
5	7.1 ± 0.009	Non‐competitive
7‐Deazaxanthine	17.3 ± 0.001	Non‐competitive

### 2.3 Molecular Docking Analysis

The Molecular Operating Environment (MOE) software performed human TP (*h*TP) and *Escherichia coli* TP (*Ec*TP) inhibition profiles and binding affinities with the ligand. The overall sequence similarity between the TP of *Ec*TP and *h*TP is ∼42%, the α‐domain is the most conserved domain in EcTP [20, 21, 22]. Amino acid residue Phe210 is not conserved in EcTP, and the rest of the active‐site amino acid residues are conserved. The residues His116, Ser117, Leu148, Arg171, Ser186, Lys190, Arg202, Val208, Ile214, His216, Lys221 and Val241 make up the active site of hTP comprehensively. The amino acid residue Phe210 in EcTP is equivalent to Val241 in hTP. Arg171, Lys190, His85, His116, and Ser186 are a few active site residues crucial to all catalytic activities [23].

To validate the docking results, docking with the 7‐Deazaxanthine was performed at the active site. Bioactive compounds isolated from *F. adenophylla* (**1–5**) are found to align with the co‐crystallized ligand, this implies that a reliable docking protocol was followed. The crystal structure of targeted proteins was obtained from Protein Data Bank in pdb format by using accession codes 2J0F and 4EAD.

**FIGURE 3:**
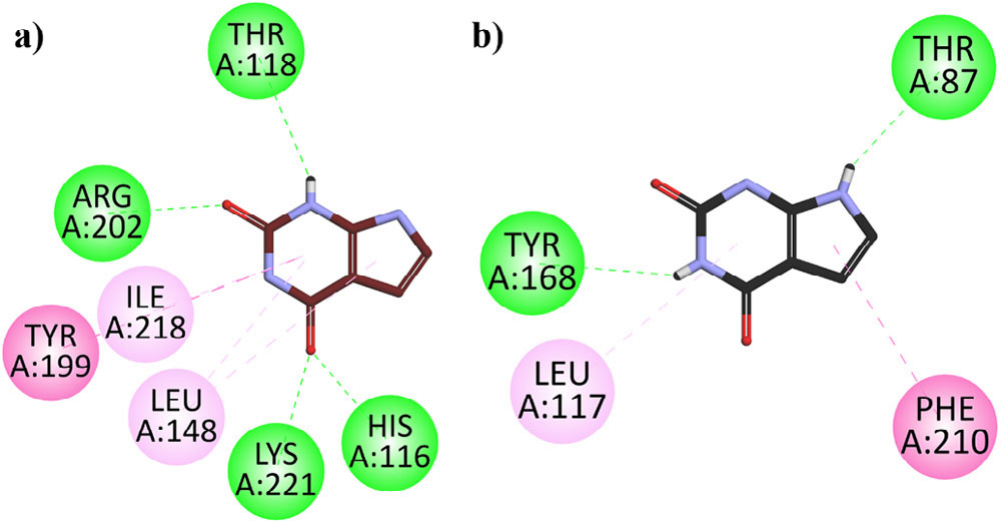
Two‐dimensional (2D) interaction plots of 7‐deazaxanthine with the binding site of *h*TP (**a**) and *Ec*TP (**b**) modeled using the Discovery Studio Visualizer (DSV).

Two‐dimensional (2D) interaction plots of *h*TP in the binding site of *h*TP and *Ec*TP in the binding site of (*Ec*TP) against 7‐deazaxanthine are shown in Figure 3a,b. 7‐Deazaxanthine showed good interactions such as conventional H‐bonds, π‐π T‐shaped, π‐Alkyl Hydrophobic, etc. with the *h*TP and *Ec*TP. The binding energy values computed for 7‐deazaxanthine in the binding site of *h*TP and *Ec*TP are ‐6.52 and ‐6.41 kcal/mol, respectively.

**FIGURE 4:**
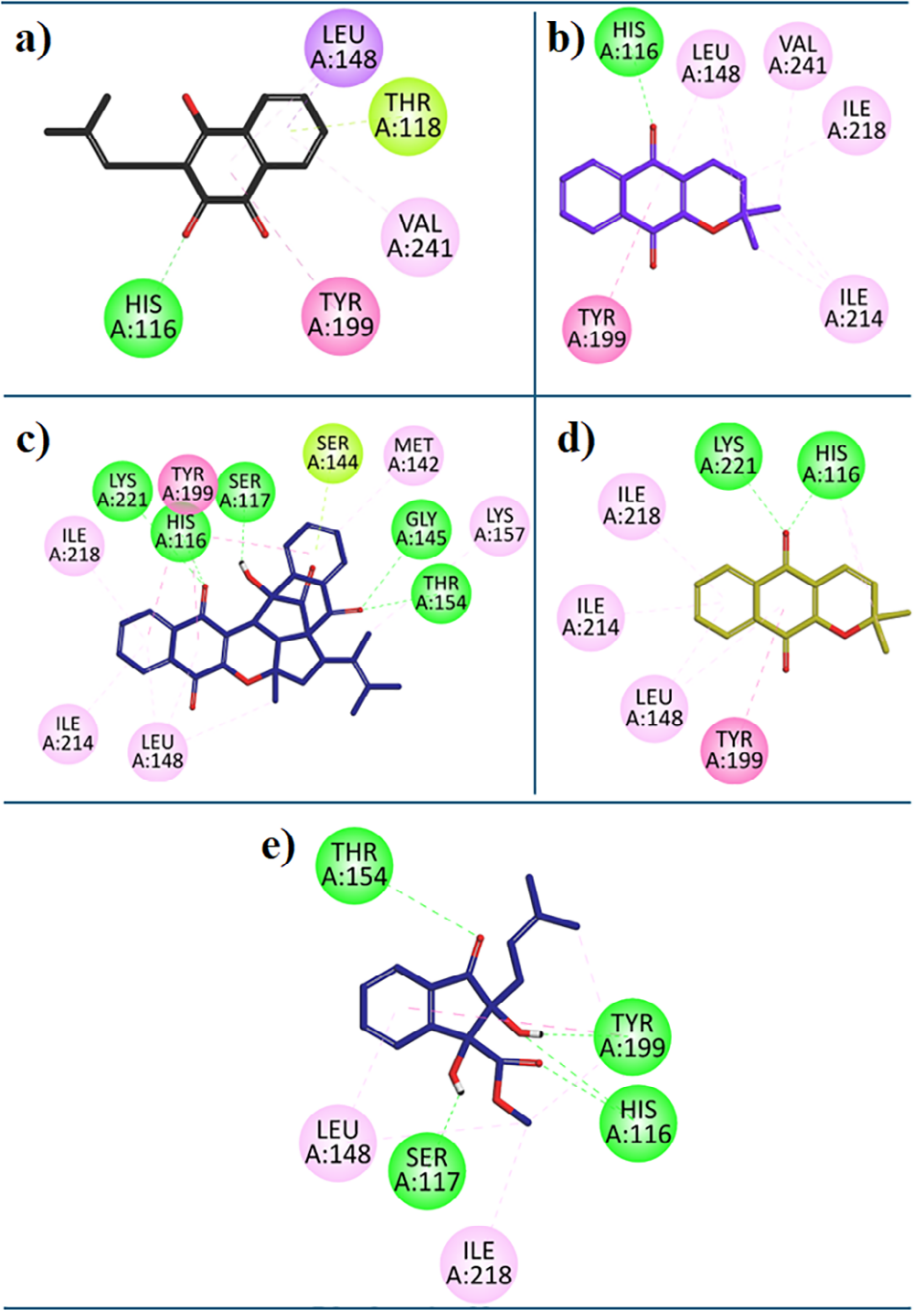
(**a**) 2D Interaction plots of the bioactive moieties are isolated from *F. adenophylla*: Lapachol (**1**), (**b**) Alpha‐Lapachone (**2**), (**c**) Peshawaraquinone (**3**), (**d**) Dehydro‐alpha‐lapachone (**4**), and (**e**) Indanone derivative (**5**) in the binding site of hTP modeled using the Discovery Studio Visualizer (DSV).

7‐Deazaxanthine showed good interaction with the *h*TP protein (Figure 3a). Lapachol (**1**) expressed diverse and very good inhibition interactions with the *h*TP enzyme. Lapachol interacts with the *h*TP receptor through conventional hydrogen bonds with residue HIS:116, π‐ π T‐shaped interaction with residue TYR:199, π‐Sigma interaction with LEU:148, π‐alkyl hydrophobic interaction with VAL:241 and π‐lone pair interaction with THR:118 (Figure 4a). In Figure 4b, the 2D interaction plot of Alpha‐Lapachone (**2**) in the *h*TP enzyme active site is shown. α‐Lapachone (**2**) uttered very good inhibition interactions with the *hTP*‐targeted protein. α‐Lapachone interacts with the *h*TP receptor through conventional hydrogen bonds with residue HIS:116, π‐π T‐shaped interaction with residue TYR:199, π‐Alkyl hydrophobic interaction with LEU:148 and Alkyl Hydrophobic interactions with residues VAL:241, LEU:148, ILE:218 and ILE:214 (Figure 4b). Figure 4c shows the 2D plot of the Peshawaraquinone (**3**) with the binding pocket of the *h*TP enzyme. Peshawaraquinone showed very versatile and comprehensive inhibition pattern with *h*TP enzyme such as five Conventional Hydrogen Bonds interactions with residues GLY:145, THR:154, SER:117, HIS:116, and LYS:221, Alkyl hydrophobic interaction with residues LYS:157, π‐Lone Pair interactions with residue SER:144, hydrophobic π‐Alkyl interactions with residues ILE:218, ILE:214, LEU:148, MET:142 and with LYS:157, π‐π T‐shaped interaction with residues TYR:199. Dehydro‐alpha‐lapachone (**4**) showed very good hydrophobic and hydrophilic interactions with the *h*TP enzyme. Dehydro‐alpha‐lapachone showed two Conventional Hydrogen Bond interactions with residues LYS:221 and HIS:116, three π‐Alkyl interactions with residues ILE:218, ILE:214, LEU:148, and a π‐π T‐shaped interactions with residues TYR:199 (Figure 4d). An important isolated compound Indanone derivative (**5**) showed very good inhibition interactions with the *h*TP enzyme. Indanone derivatives interact with the *h*TP receptor via five conventional hydrogen bond interactions with residues THR:154, SER:117, HIS:116, and TYR:119, three hydrophobic π‐Alkyl interactions with residues ILE:218, LEU:148, and a π‐π T‐shaped interactions with residues TYR:199 (Figure 4e).

**FIGURE 5:**
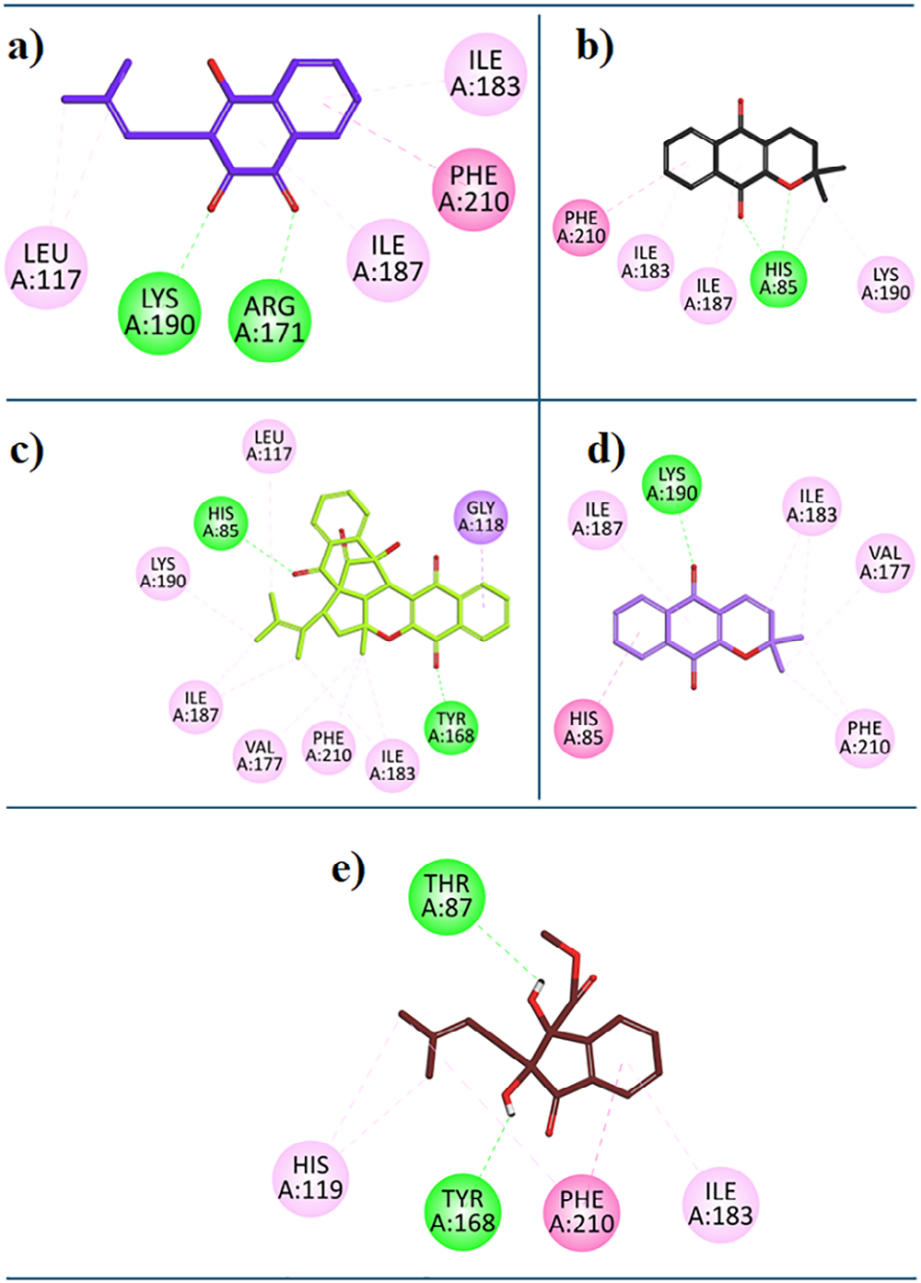
**a**) 2D Interaction plots of the bioactive moiety isolated from *F. adenophylla*: Lapachol (**1**), **b**) Alpha‐Lapachone (**2**), **c**) Peshawaraquinone (**3**), **d**) Dehydro‐alpha‐lapachone (**4**) and **e**) Indanone derivative (**5**) in the binding site of EcTP modeled using the Discovery Studio Visualizer (DSV).

For the current study, we have performed the docking study with the binding site of *Ec*TP. Referenced compound 7‐Deazaxanthine showed good interaction with the active site of the *Ec*TP protein. Lapachol (**1**) expressed diverse inhibition interactions with the *Ec*TP enzyme. Lapachol interacts with the *Ec*TP receptor through two Conventional Hydrogen Bonds with residue LYS:190, a π‐ π T‐shaped interaction with residue PHE:210, Alkyl hydrophobic interaction with residue LEU:117, two hydrophobic π‐Alkyl interactions with residues ILE:183, ILE:187 (Figure 5a). In Figure 5b, the 2D interaction plot of Alpha‐Lapachone (**2**) in the *Ec*TP enzyme active site is shown. α‐Lapachone (**2**) expressed good inhibition interactions with the *EcTP‐targeted* protein. α‐Lapachone interacts with the *Ec*TP receptor through two Conventional Hydrogen Bonds with residue HIS:85, π‐ π T‐shaped interaction with residue PHE:210, two π‐Alkyl hydrophobic interactions with ILE:183, ILE:187, and an alkyl hydrophobic interaction with residue LYS:190 (Figure 5b). Figure 5c shows the 2D plot of the Peshawaraquinone (**3**) with the binding pocket of the *Ec*TP enzyme. Peshawaraquinone showed versatile and comprehensive inhibition patterns with *Ec*TP enzyme such as; two Conventional Hydrogen Bonds with residue HIS:85 and TYR:168, π‐ Sigma interaction with residue GLY:118, a π‐Alkyl hydrophobic interactions with PHE:210, and five Alkyl Hydrophobic interaction with residue LYS:190, LEU:117, ILE:183, ILE:187, VAL:177. Dehydro‐alpha‐lapachone (**4**) showed good hydrophobic and hydrophilic interactions with the *Ec*TP enzyme. Dehydro‐alpha‐lapachone showed a Conventional Hydrogen Bond with residue LYS:190 and, π‐π T‐shaped interaction with residue HIS:85, a π‐Alkyl hydrophobic interactions with PHE:210, ILE:187, and two Alkyl Hydrophobic interactions with residue ILE:183, VAL:177 (Figure 5d). An important isolated compound Indanone derivative (**5**) showed good inhibition interactions with the *Ec*TP enzyme. Indanone derivative interacts with *Ec*TP receptor via two conventional hydrogen bonds with residues THR:87, TYR:168, and π‐ π T‐shaped interaction with residue PHE:210, two π‐Alkyl hydrophobic interactions with HIS:119, ILE:183 (Figure 5e).

## 3 Discussion

Medicinal plants have long been recognized as valuable sources of bioactive compounds [24, 25], which have played a pivotal role in the development of novel therapeutic agents in modern pharmacology [26, 27, 28]. Natural products derived from plants exhibit a diverse array of chemical structures and possess a myriad of biological activities, making them indispensable reservoirs for drug discovery and development [29, 30]. The rich biodiversity of plants offers a vast repertoire of phytochemicals, each with unique pharmacological properties and therapeutic potential. These bioactive compounds, derived from medicinal plants, encompass a wide range of activities, including antioxidant, anti‐inflammatory, antimicrobial, antiviral, anticancer, and immunomodulatory effects [31]. In the context of this study, the investigation into the TP inhibitory potential of bioactive compounds isolated from *F. adenophylla* exemplifies the significance of medicinal plants in providing leads for the development of targeted therapeutics [27]. The findings underscore the pharmacological importance of these natural products and their potential utility in addressing TP‐related pathologies, thereby emphasizing the ongoing relevance of medicinal plants in modern drug discovery endeavors. Through the integration of in vitro assays and in silico studies, this research not only elucidates the therapeutic potential of bioactive compounds from *F. adenophylla* but also underscores the broader significance of natural products in pharmaceutical research, providing insights into their molecular mechanisms of action and guiding the rational design of novel pharmacological agents. The investigation into the TP inhibitory potential of bioactive compounds isolated from *F. adenophylla* presents promising avenues for therapeutic intervention in various pathologies. The results of the in vitro assays revealed significant TP inhibitory activity among the isolated compounds, indicating their potential as effective inhibitors of TP enzyme activity. Lapachol (**1**), Alpha‐lapachone (**2**), Peshawaraquinone (**3**), and Indanone derivative (**5**) demonstrated notable inhibition of TP, with inhibition percentages ranging from 86.2% to 91.3%. These findings suggest the pharmacological significance of these compounds in targeting TP‐related pathologies. The observed differences in the inhibitory activity among the compounds, as evidenced by their IC_50_ values, provide insights into their potency in inhibiting TP activity. Lapachol (**1**) and Indanone derivative (**5**) exhibited particularly promising IC_50_ values, indicating their efficacy in inhibiting TP at relatively low concentrations. Conversely, dehydro‐α‐lapachone (**4**) showed comparatively lower inhibitory activity, suggesting variations in the effectiveness of TP inhibition among the isolated compounds. The inclusion of 7‐deazaxanthine as a standard inhibitor provided a basis for comparison and validation of the results. Despite exhibiting notable inhibition of TP activity, 7‐deazaxanthine displayed higher IC_50_ values compared to Lapachol (**1**) and Indanone derivative (**5**), highlighting the potential superiority of these compounds as TP inhibitors.

We have performed docking studies of bioactive compounds isolated from *F. adenophylla* against receptors *h*TP and *Ec*TP for the current study. Both the receptors (hTp and *Ec*Tp) played similar roles including cancer, inflammation, and angiogenesis, and are very much conserved. The docking protocol used in this study was validated by docking the co‐crystallized ligand, 7‐deazaxanthine with both receptors, which shows the reliable binding profiles and confirms the accuracy of the simulation step.

Among the tested Bioactive compounds, Lapachol (**1**) demonstrated good inhibition against *h*TP and *Ec*Tp, exhibiting a good interaction profile in both enzymes. It achieved the lowest binding energy value of Lapachol, which is ‐6.91 kcal/mol for *h*TP and ‐5.48 kcal/mol for *Ec*TP suggesting that it acts as a non‐selective inhibitor of both enzymes. Taken together, the dual inhibition potential of Lapachol highlights its therapeutic relevance in targeting TP from both human and bacterial sources. Moreover, a comparative analysis of in vitro and in silico findings reinforces this conclusion and provides mechanistic insight. Bioactive compound Alpha‐lapachone (**2**) also exhibits moderate inhibitory activity against both enzymes. Its binding energy S values are ‐6.47 kcal/mol and ‐5.60 kcal/mol for *h*TP and *Ec*TP, respectively indicating that while it is effective, it may not be as so good as (**1**). The interactions observed suggest that (**2**) could be a promising lead compound. Another bioactive compound isolated from *F. adenophylla*, Peshawaraquinone (**3**) is found to actively and efficiently inhibit the *h*TP and *Ec*TP enzymes with binding energy S value ‐6.26 and ‐5.12 kcal/mol, respectively. This compound displayed the versatile interaction profile and engaged the *h*Tp and *Ec*Tp in multiple binding modes. Dehydro‐α‐lapachone (**4**) showed good inhibition interaction with both enzymes with binding energy S values ‐6.13 kcal/mol and ‐5.5 kcal/mol for *h*TP and *Ec*TP, respectively. Although its binding affinities were lower as compared to the (**3**), it still displayed strong interaction characteristics and can be considered further to enhance its efficacy. Lastly, Indanone derivative (**5**), the bioactive compound isolated from *F. adenophylla*, showed good inhibition interactions with both receptors. It interacts with *h*TP through five conventional hydrogen bond interactions, three hydrophobic π‐alkyl interactions, and a π‐π T‐shaped interaction and interacts with *Ec*TP via two conventional H‐bonds, π‐ π T‐shaped interactions, two π‐Alkyl hydrophobic interactions. The lowest binding energy S values of Indanone derivative (**5**) are ‐7.5 and ‐7.8 kcal/mol for *h*TP and *Ec*TP, respectively. This compound was found to be a potent dual target (*h*Tp and *Ec*Tp) inhibitor. The in silico molecular docking analysis supported the in vitro results by elucidating the binding interactions between isolated compounds and the active site of the TP enzyme, identifying key residues and binding modes. The binding energy values computed for the standard drug used in the in‐vitro assay (7‐Deazaxanthine) in the binding site of *h*TP and *Ec*TP are ‐6.52 and ‐6.41 kcal/mol, respectively. This combined approach enhances our understanding of TP inhibition and informs the rational design of TP‐targeted therapeutics. These correlations support their non‐competitive inhibition profile and validate the experimental outcomes. Conversely, dehydro‐α‐lapachone (**4**), with weaker inhibition (IC_50_ = 17.2 µM), displayed less stable docking poses and fewer key interactions, further confirming the reliability of the integrated approach.

The current findings substantially advance prior work on natural TP inhibitors, particularly the study by Javaid et al., who screened 18 natural compounds and reported moderate to weak TP inhibition, with IC₅₀ values ranging from 44.0 to 420.3 µM. In contrast, the present study identified several compounds, including Lapachol (**1**), alpha‐lapachone (**2**), and an indanone derivative (**5**), with significantly more potent TP inhibitory activity (IC₅₀ = 1.8–3.6 µM), suggesting superior binding affinity and therapeutic potential [18]. The variation in TP inhibitory activity among the isolated compounds may be attributed to differences in functional groups and substitution patterns on the core structures. Certain substituents likely enhance binding through hydrogen bonding or electronic effects, while others may interfere with optimal interactions due to steric hindrance. These observations suggest that small structural changes can significantly impact biological activity, highlighting key features for future optimization of TP inhibitors.

The binding energy values computed for the standard drug used in the in‐vitro assay (7‐deazaxanthine) in the binding site of *h*TP and *Ec*TP are ‐6.52 and ‐6.41 kcal/mol, respectively (Table 3). This combined approach enhances our understanding of TP inhibition and informs the rational design of TP‐targeted therapeutics. The findings highlighted the therapeutic potential of bioactive compounds from *F. adenophylla* as TP enzyme inhibitors, meriting further preclinical and clinical studies to confirm their efficacy and safety for clinical use.

**TABLE 3:** Docking score (binding energy) and target enzymes for bioactive compounds isolated from *F. adenophylla* against human thymidine phosphorylase (*h*TP) and *Escherichia coli* TP (*Ec*TP).

Sr.#	Compound name	Binding energies (*h*TP) (kcal/mol)	Binding energies (*Ec*TP) (kcal/mol)
1.	Lapachol	−6.91	−5.48
2.	Alpha‐Lapachone	−6.47	−5.60
3.	Peshawaraquinone	−6.26	−5.12
4.	Dehydro‐α‐lapachone	−6.13	−5.50
5.	Indanone Derivative	−7.50	−7.80
6.	7‐Deazaxanthine	−6.52	−6.41

Despite the promising enzyme inhibitory and in silico results of the isolated compounds against TP, our study has certain limitations. Firstly, the biochemical assays were conducted using Escherichia coli TP, which, although commonly used as a model enzyme, may differ structurally and functionally from the mammalian counterpart. These interspecies differences might affect the binding affinity and inhibitory mechanism of the test compounds. Secondly, while our in vitro and computational findings suggest potential anti‐angiogenic properties, the absence of in vivo studies limits the translation of these results to physiological systems. Future studies involving mammalian TP and in vivo models will be essential to validate the therapeutic relevance, bioavailability, pharmacokinetics, and toxicity profiles of the most active compounds.

The findings highlighted the therapeutic potential of bioactive compounds from *F. adenophylla* as TP enzyme inhibitors, meriting further in vivo, preclinical, and clinical studies to confirm their efficacy and safety for clinical use.

## 4 Materials and Methods

### 4.1 Plant Collection

The stem of *F. adenophylla* was collected from Peshawar, Pakistan in July 2023. Authentication was conducted at the Department of Botany, University of Swabi, by a plant taxonomist Dr, Mohammad Ilyas. A voucher specimen (UOS/Bot761) was deposited in the herbarium for reference.

### 4.2 Extraction and Isolation of Compounds

**FIGURE 1:**
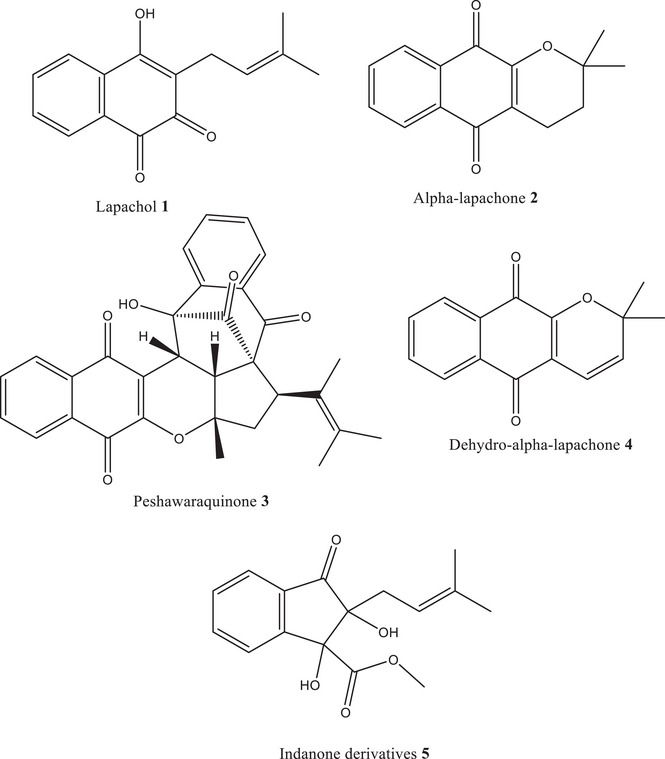
Chemical structures of compounds isolated from *F. adenophylla*.

Following meticulous collection, the harvested stems of *F. adenophylla*, weighing a total of 10 kg, underwent a careful drying process under shaded conditions for 15 days. This meticulous drying process ensured the preservation of the plant's bioactive constituents while minimizing the risk of degradation. Once adequately dried, the stems were finely ground into a powder, thereby facilitating efficient extraction of the desired compounds. The extraction process commenced with a normal cold extraction method. After extraction, the crude extract was fractionated using solvents of increasing polarity: n‐hexane, dichloromethane, ethyl acetate, and methanol. The fractions were evaluated by thin‐layer chromatography (TLC) to determine the presence of bioactive compounds. Based on its TLC profile, the methanolic fraction was selected for further purification. Based on its TLC profile, the methanolic fraction was selected for subsequent purification using normal phase column chromatography involves the passage of the methanolic fraction through a stationary phase packed in a column. Through this systematic purification process, the bioactive compounds, that is, Lapachol (**1**), alpha‐lapachone (**2**), Peshawaraquinone (**3**), dehydro‐α‐lapachone (**4**), and indanone derivative (**5**) were successfully isolated from the methanolic fraction. The structure of these compounds was previously identified by our research group [31, 32]. The structure of these compounds is given in the inset of Figure 1.

### 4.3 TP Assay

In this study, we utilized commercially available recombinant *Ec*TP enzyme as a model for TP inhibition assessment due to its structural similarities with mammalian TP enzymes. Spectrophotometric TP inhibition assays were conducted by incubating 0.058 U of *Ec*TP enzyme with 500 µM of test compounds at 30°C for 10 min. Subsequently, 1.5 mM of thymidine substrate was added, and absorbance changes were monitored for 10 min at 290 nm using a microplate reader. All experiments were performed in triplicate, with 7‐deazaxanthine as the standard inhibitor. IC_50_ values were determined by preparing a series of concentrations for each compound and calculating the concentration required to inhibit 50% of TP activity. Mechanistic studies involved incubating the TP enzyme with varying inhibitor concentrations and substrate concentrations ranging from 0.1875 to 1.5 µM. Enzyme activity was assessed by measuring absorbance changes for an additional 10 min. Triplicate experiments were conducted for mechanistic investigations to ensure the results' robustness [18].

### 4.4 Kinetic Studies

Chemical reactions were examined using kinetic studies to determine their rates, mode of inhibition, and the variables influencing these characteristics [33, 34]. The kind and mechanism of inhibition of derivatives are ascertained here. Test compounds (0.5–500 µM) were incubated with TP (0.058 U/200 µL) for 10 min at 30°C in mechanistic studies. The addition of various thymidine concentrations (varying from 0.1875 to 1.5 µM) then started the process. Thymidine cleavage was tracked at 290 nm for ten min straight using an ELISA plate reader (SpectraMax 384; Molecular Devices, San Jose, CA, USA).

### 4.5 Molecular Docking

To investigate the inhibition interactions of bioactive compounds isolated from *F. adenophylla* (**1–5**) and TP the MOE software version 2016.08 is used along with Discovery Studio Visualizer (DSV) v24.1.0.23298 for interaction analysis and MOE output interpretation. In our current study, TP of two different organisms, for example, Homo sapiens (*H. sapiens*) and *E. coli* K‐12 is used with the expression system Escherichia coli and Escherichia coli BL21(DE3), respectively. Crystal structure of *h*TP and *Ec*TP were obtained from Protein Data Bank (PDB) with accession codes of 2J0F and 4EAD, respectively. The crystal structure of 2J0F is a non‐trypsinized *h*TP thymine complex. While the crystal structure of *Ec*TP is complex with 2',3'‐dideoxy‐2'‐fluoro‐3'‐triaza‐1,2‐dien‐2‐ium‐1‐yluridine. The TP enzyme's structural investigation showed that the phosphate, thymine, and thymidine binding sites are in a cavity‐like region that is 8Å wide and 10Å deep [35]. The crystal structures of the TP enzymes are downloaded, and after analyzing the sequence the water molecules are removed, and energy is optimized by QuickPrep (energy minimization using Amber10EHT force field, protonate three‐dimensional [3D] at 300 K and 7.0 pH) in the MOE interface using the default Force Field and RMS Gradient of 0.1 from the receptor protein structure.

The 3D structures of compounds (**1–5**) were modeled with the help of the builder program of MOE. MOE's Protonate 3D tool was used to assign the most probable protonation states, and tautomeric forms according to physiological pH (7.0). After 3D protonation, each molecule was energy minimized using force field Amber10: EHT; R‐Field 1:80; Cutoff in the gas phase solvation with the gradient of 0.00001 kcal/mol. The minimized ligands were saved in MOE database (.mdb) format for subsequent docking.

After the preparation of the downloaded enzymes, validation of the docking protocol via redocking was carried out by comparing the conformation/orientation of native ligands and docked poses. The procedure with root‐mean‐square deviation less than 2 Å was used for docking of compounds.

The binding site for docking was defined as all protein residues within 10 Å of the co‐crystallized ligand. Pose generation was performed with the Triangle Matcher placement method and then scored initially by the London dG function. All poses were subsequently refined using force field‐based energy minimization, and binding affinities were estimated by using the GBVI/WSA dG rescoring function. For each ligand, docking poses were generated and stored in numbers ranging between 10 and 30. and the ligands' binding energy was ranked through LondonDG and GBVI/WSAdG scoring measured by unit kcal/mol. After docking, the lowest energy pose of the ligand is used for further analysis. To analyze the 2D and 3D interactions MOE and DSV software programs were used.

### 4.6 Statistical Analysis

Statistical analysis was conducted to assess the significance of the experimental data obtained from the TP inhibition assays and mechanistic studies. The findings of this study are presented as mean ± standard error of the mean to determine statistically significant differences (*p* < 0.05 or 0.01). Statistical analysis was performed using GraphPad Prism software.

## 5 Conclusion

The results of this study show the significant potential of compounds isolated from *F. adenophylla* as effective inhibitors of TP, highlighting the importance of medicinal plants in drug discovery and development. Through a combination of in vitro assays and molecular docking analysis, we identified Lapachol (**1**), alpha‐lapachone (**2**), Peshawaraquinone (**3**), dehydro‐α‐lapachone (**4**), and indanone derivative (**5**) as promising TP inhibitors, showing good activity. The docking analysis, supported by *in vitro* analysis, showed that compounds **1** and **5** are found dual‐target inhibitors to the TP in both human and bacterial systems. These bioactive compounds isolated from *F. adenophylla* offer new avenues for the development of TP‐targeted therapeutics, with implications for the treatment of various TP‐related pathologies, including cancer and inflammation. The comprehensive approach employed in this study, integrating experimental and computational techniques, enhances our understanding of the molecular mechanisms underlying TP inhibition and facilitates the rational design of future drug candidates. Moving forward, further research should focus on elucidating the pharmacological profiles and therapeutic potential of these compounds in vivo, paving the way for their translation into clinically viable treatments.

## Author Contributions

**Abdur Rauf**: conceptualized and supervised the study, and wrote the original draft. **Marcello Iriti**: conceptualized and supervised the study, and wrote the original draft. **Majid Khan**: performed the phytochemical isolation and characterization, and wrote the original draft. **Zubair Ahmad**: performed the phytochemical isolation and characterization. **Umer Rashid**: conducted the molecular docking and computational studies. **Zafar Ali Shah**: conducted the molecular docking and computational studies. **Anees Saeed**: conducted the molecular docking and computational studies. **Abdulhakeem S. Alamri**: carried out the enzyme inhibition assays. **Walaa F. Alsanie**: carried out the enzyme inhibition assays. **Imtiaz Khan**: carried out the enzyme inhibition assays. **Humaira Hussain**: contributed to data analysis and manuscript editing. **Majid Alhomrani**: contributed to data analysis and manuscript editing. All authors reviewed and approved the final version of the manuscript.

## Conflicts of Interest

The authors declare no conflicts of interest.

## Data Availability

The data associated with this paper is available with the corresponding authors upon request.
